# Case report: A rare case of triple negative breast cancer with development of acute pancreatitis due to dexamethasone during adjuvant chemotherapy

**DOI:** 10.3389/fonc.2024.1340419

**Published:** 2024-02-15

**Authors:** Hirofumi Ohmura, Taro Tobo, Yuki Ando, Takaaki Masuda, Koshi Mimori, Koichi Akashi, Eishi Baba

**Affiliations:** ^1^ Department of Oncology and Social Medicine, Kyushu University Graduate School of Medical Sciences, Fukuoka, Japan; ^2^ Department of Clinical Laboratory Medicine, Kyushu University Beppu Hospital, Beppu, Japan; ^3^ Department of Surgery, Kyushu University Beppu Hospital, Oita, Japan; ^4^ Department of Medicine and Biosystemic Science, Kyushu University Graduate School of Medical Sciences, Fukuoka, Japan

**Keywords:** breast cancer, adjuvant-chemotherapy, pancreatitis, dexamethasone, adverse effects

## Abstract

Here, we present the case of a 42-year-old female who developed acute pancreatitis due to dexamethasone during adjuvant chemotherapy for early triple negative breast cancer (TNBC). The patient received partial mastectomy and sentinel lymph node biopsy for early TNBC (cT1N0M0, cStage I) of the left breast. Dose-dense doxorubicin plus cyclophosphamide (ddAC) was administered as the adjuvant-chemotherapy; however, epigastralgia appeared on the fifth day of the first administration. A blood test showed a remarkable increase of serum pancreatic enzyme levels and computed tomography (CT) showed the swelling of pancreas and surrounding effusion, and she was diagnosed with moderate acute pancreatitis. As she had no history of excessive alcohol consumption or complication of cholelithiasis, dyslipidemia, or pancreatic neoplasm, drug-induced pancreatitis was suspected. Dexamethasone, which was administered as an antiemetic, was the suspected drug based on the drug administration history and previous report, and dexamethasone was discontinued from the second administration of ddAC. There was subsequently no recurrence of pancreatitis with no increase in serum pancreatic enzyme levels, and it was possible to complete adjuvant-chemotherapy. Alcohol, gallstones, dyslipidemia, and drugs have been reported as causes of pancreatitis; however, steroid-induced acute pancreatitis is extremely rare. We present the first case of acute pancreatitis induced by dexamethasone as the antiemetic.

## 1 Introduction

Breast cancer is the most common malignancy and a leading cause of cancer death in women worldwide (1). Early-stage breast cancer is treated with surgery, while multimodal therapy including radiation, perioperative chemotherapy is added according to the risk factors for recurrence. In operable breast cancer, adjuvant chemotherapy and neoadjuvant chemotherapy have shown no significant difference in overall survival (OS) and disease-free survival (DFS) (2). Pathologic complete response after neoadjuvant chemotherapy is associated with improved long-term outcomes (3–5). Standard perioperative chemotherapy options for HER2-negative breast cancer include anthracyclines and taxanes. A typical regimen is 4 cycles of doxorubicin plus cyclophosphamide (AC) followed by 4 cycles of paclitaxel (PTX). Compared to standard 3-week cycles, dose-dense chemotherapy with the support of granulocyte colony-stimulating factor and 2-week cycles significantly improved DFS and OS (6).

Acute pancreatitis is diagnosed based on at least two of the following revised Atlanta criteria: (1) abdominal pain suggestive of pancreatitis, (2) serum amylase or lipase >3 times the upper limit of normal, and (3) imaging findings of acute pancreatitis on CT or MRI (7, 8). Common causes are gallstones and alcohol, while genetic and drug-induced are less common. The pathogenesis involves damage to pancreatic acinar cells, inappropriate intra-acinar trypsinogen activation, and autodigestion of the pancreatic parenchyma. Activated pancreatic enzymes injure the pancreatic tissue, inducing inflammatory cytokines through NFκB signaling, resulting in inflammation and edema of the pancreas. In gallstone pancreatitis, increased pancreatic duct pressure from obstructed outflow is thought to be the main cause, while the pathogenesis of alcohol-induced pancreatitis has not been well elucidated (9). Genetic mutations have been identified as the cause of pancreatitis, including *PRSS1* mutations in cationic trypsinogen, and *SPINK1* mutations associated with inactivation of trypsin (10). Drug-induced pancreatitis is rare; however, more than 100 medications have been reported to be associated with pancreatitis. Direct toxicity for pancreas, accumulation of metabolites, ischemia of pancreatic tissue, and increased viscosity of pancreatic secretions have been suggested as the mechanisms for the onset of drug-induced pancreatitis; however, the mechanisms underlying drug-induced pancreatitis have not been well elucidated for most causative drugs (11, 12).

## 2 Case description

**Figure 1:**
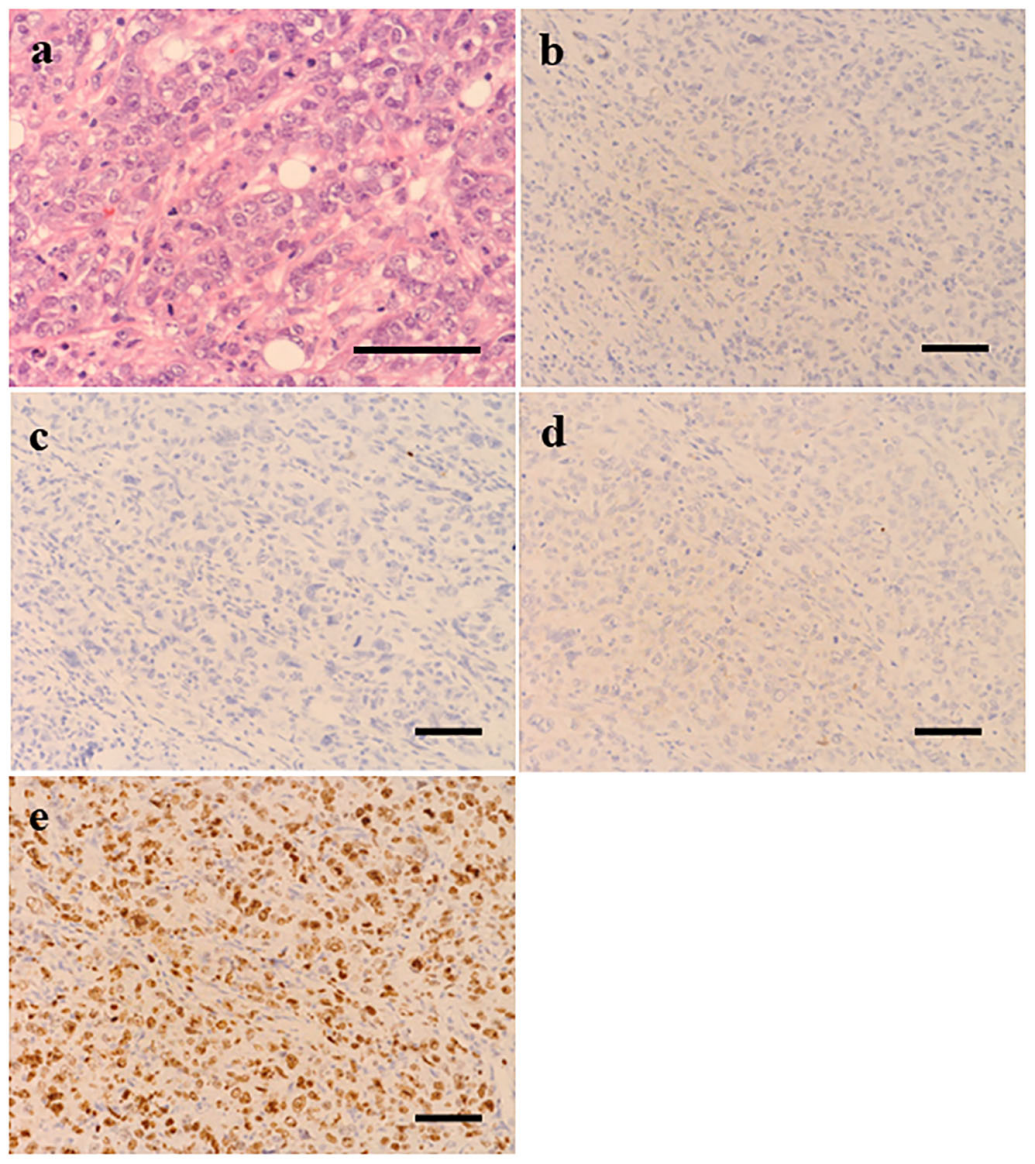
Histopathological examination (scale bar 50 μm) of the surgical specimen from the lesion of left breast showed invasive ductal carcinoma grade 3 **(A)**. Immunohistochemically, tumor cells were negative for estrogen receptor **(B)**, progesterone receptor **(C)** and HER2 **(D)** and Ki67 score was 78% **(E)**.

**Figure 2:**
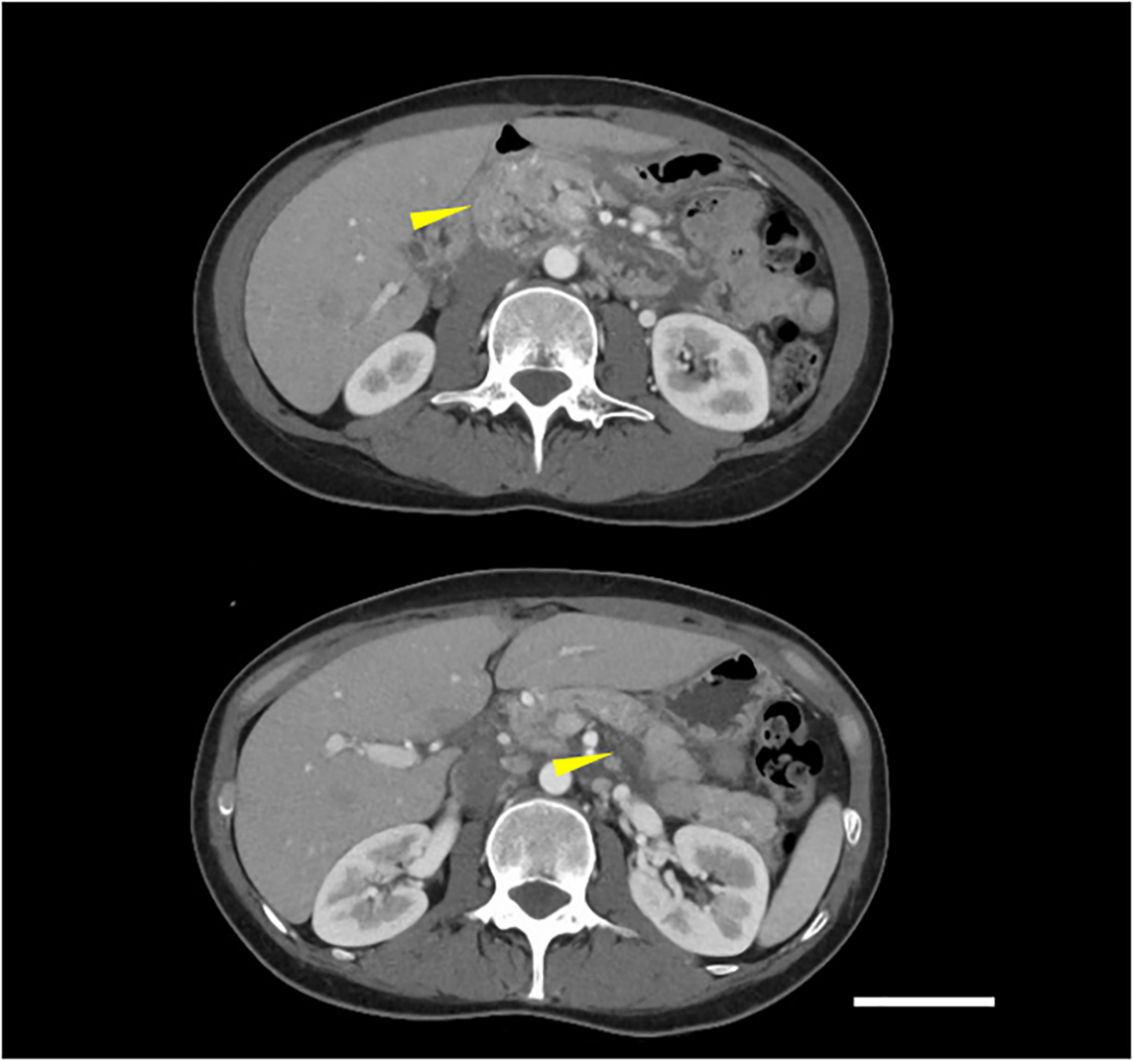
Contrast-enhanced CT showed pancreatic enlargement and peri-pancreatic fluid (indicated by yellow arrows), meeting criteria for mild acute pancreatitis according to the revised Atlanta classification (scale bar 5 cm).

**Figure 3:**
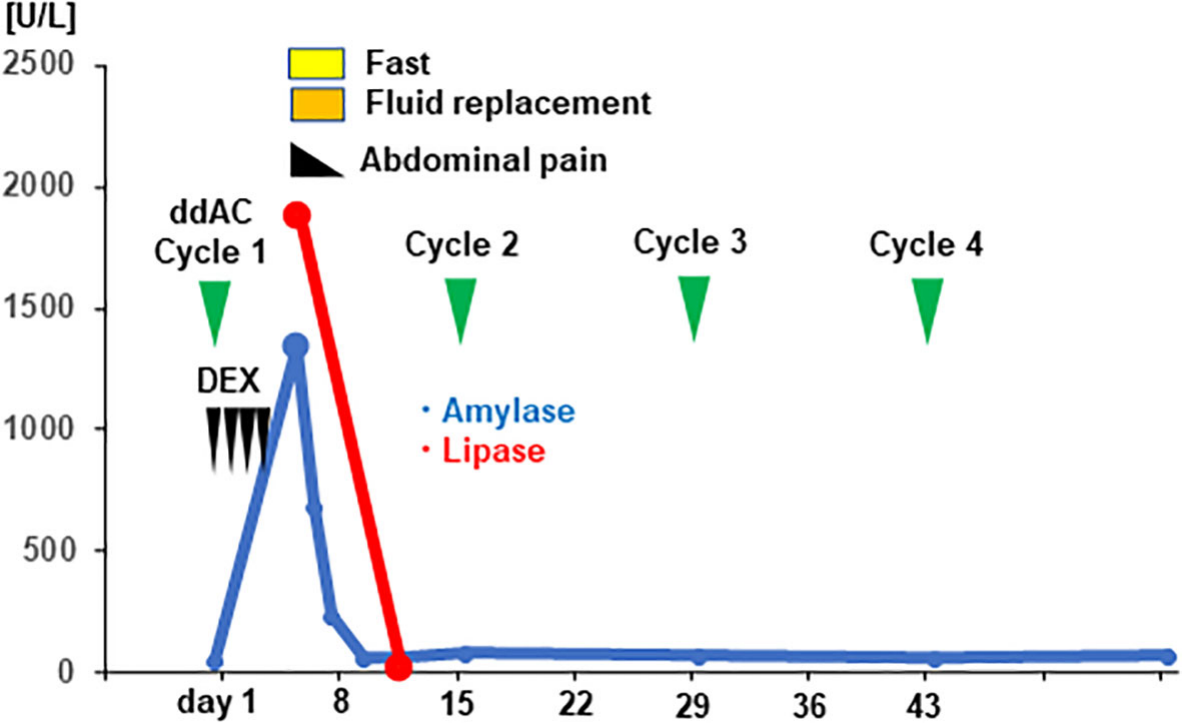
Clinical time course and the serum level of pancreatic enzymes of the patient are shown. The vertical axis of the graph shows the serum levels of pancreatic enzymes [U/mL], and the horizontal axis shows the days from initiation of dose-dense doxorubicin plus cyclophosphamide (ddAC). Dexamethasone (DEX) was administered on day1-4.

A 42-year-old woman presented to a local clinic in March 2019 with a complaint of left breast mass with the longest diameter of 15mm in the C region by ultrasound examination. The patient had undergone breast cancer screening in the previous year, and mammography revealed no abnormal findings. Core needle biopsy from the left breast mass showed invasive ductal carcinoma, solid tubular type, tumor size; invasive area 18×16mm, nuclear grade (NG) 3, hormone receptor (estrogen receptor, ER; and progesterone receptor, PgR) negative, and HER2 negative. The patient was referred to our surgical department in April for further treatment of TNBC cT1N0M0, cStage I. The patient had no family history of pancreatic cancer or pancreatitis, or history of excessive alcohol consumption, cholelithiasis, diabetes, dyslipidemia or hypertension. And she had no medication or food allergy. In May, partial mastectomy and sentinel lymph node biopsy were performed. Postoperative pathological diagnosis revealed TNBC, T1cN0, ly0, v0, NG3, ER (Allred score 0), PgR (Allred score 0), HER2 (IHC score 0), and MIB1 (78%) (**Figures 1A–E**). Postoperative dose-dense doxorubicin plus cyclophosphamide (ddAC; doxorubicin 60 mg/m2 day1, cyclophosphamide 600 mg/m2 day1, 2-week cycle)×4 cycles followed by dose-dense paclitaxel (ddPTX; paclitaxel 175 mg/m2 day1, 2-week cycle)×4 cycles, then radiation to the residual breast, was planned for the adjuvant therapy. Administration of ddAC was initiated in August. As antiemetics, dexamethasone (9.9mg/body day1, intravenous; 8mg/body day2-4, oral), aprepitant (125mg/body day1; 80mg/body day2-3, oral), and granisetron (3mg/body, day1, intravenous) were administered. On day 5 of the first cycle of ddAC, the patient developed epigastric pain, with marked elevation of pancreatic enzymes (amylase 1344 U/L (pancreatic amylase, 97.4%; salivary amylase, 2.6%), lipase 1895 U/L). Contrast-enhanced CT showed pancreatic enlargement and peri-pancreatic fluid, meeting criteria for mild acute pancreatitis by the revised Atlanta classification (**Figure 2**). With fasting and intravenous fluid administration, pancreatic enzymes rapidly decreased, and the abdominal pain resolved (**Figure 3**). Re-examination of CT after 3 days showed the resolution of the peri-pancreatic fluid, indicating improvement of the pancreatitis. The patient had no history of heavy alcohol intake, gallstones, dyslipidemia, or pancreatic tumor, implicating drug-induced pancreatitis. Among the medications, doxorubicin, cyclophosphamide, and antiemetics including dexamethasone, aprepitant, and granisetron were considered to be candidates for suspected drugs. There has been a previous report of acute pancreatitis due to dexamethasone, and with the Naranjo adverse drug reactions (ADR) probability scale (≥ 9 points, Definite; 5-8 points, Probable; 1-4 points, Possible; 0-3 points, Doubtful) (13), dexamethasone scored 6 indicating ‘Probable’, while other drugs scored 3 indicating ‘Possible’, and dexamethasone was considered as the suspected drug. Dexamethasone was omitted from cycle 2. The doses of other drugs remained unchanged. No further elevation of serum pancreatic enzymes or recurrence of pancreatitis was observed, allowing completion of 4 cycles of ddAC and 4 cycles of ddPTX. Steroid-induced acute pancreatitis is extremely rare, however, it should be recognized as a potential adverse event of chemotherapy.

## 3 Discussion

There is one previous case report of acute pancreatitis after administration of dexamethasone for spinal cord compression, with recurrence on re-challenge; however, the patient also had risk factors including alcohol intake (14). Khanna et al. reported a case of acute pancreatitis induced by re-administration of hydrocortisone for ulcerative colitis. Other steroids including prednisolone and cortisone acetate also may potentially cause acute pancreatitis (15). Glucocorticoids including dexamethasone have been reported to inhibit NFκB activation and suppress TNFα production of immune cells (16). As NFκB activation and inflammatory cytokine production are involved in the pathogenesis of pancreatitis (9), the mechanism of steroid-induced pancreatitis likely involves pathways other than NFκB activation. Previous reports have also suggested that steroids increase pancreatic juice viscosity and proliferate pancreatic ductal epithelium, delaying excretion and contributing to pancreatic autodigestion (12, 17). However, the detailed mechanisms of steroid-induced pancreatitis have not been elucidated. The association between side effects and suspected drug can be evaluated using the Naranjo ADR probability scale (13), and in this case, dexamethasone scored higher than other drugs, suggesting a stronger association with pancreatitis. By excluding gallstones, alcohol, and other common causes of pancreatitis, and based on the Naranjo score, dexamethasone-induced pancreatitis was diagnosed. In the previous case-control study by Sadr-Azodi et al., oral glucocorticoid (betamethasone and prednisolone) administration was associated with an increased risk of acute pancreatitis (OR, 1.53; 95% CI, 1.27-1.84) and the risk was highest 4 to 14 days after drug administration (OR, 1.73; 95% CI, 1.31-2.28). Dexamethasone had not been studied in this study, however, it should also be considered as a potential cause of pancreatitis as an oral glucocorticoid (18).

Although there has been one case report of pancreatitis caused by dexamethasone administered for spinal cord compression, no previous report of pancreatitis caused by dexamethasone administered for supportive therapy for chemotherapy. In this case, dexamethasone was considered to be the cause of acute pancreatitis, and by omitting dexamethasone, it was possible to complete perioperative chemotherapy for the present patient.

## 4 Conclusion

Although steroid-induced acute pancreatitis is extremely rare, it should be recognized as a potential adverse event observed during cancer chemotherapy due to use of dexamethasone as antiemetic.

## Data availability statement

The original contributions presented in the study are included in the article/supplementary material. Further inquiries can be directed to the corresponding author.

## Ethics statement

The studies involving humans were approved by Ethics Committee of Kyushu University Hospital. The studies were conducted in accordance with the local legislation and institutional requirements. Written informed consent for participation was not required from the participants or the participants’ legal guardians/next of kin in accordance with the national legislation and institutional requirements. Written informed consent was obtained from the individual(s) for the publication of any potentially identifiable images or data included in this article. Written informed consent was obtained from the participant for the publication of this case report.

## Author contributions

HO: Conceptualization, Writing – original draft, Writing – review & editing. TT: Writing – review & editing. YA: Writing – review & editing. TM: Writing – review & editing. KM: Writing – review & editing. KA: Writing – review & editing. EB: Writing – original draft, Writing – review & editing.
